# Targeting Mitochondria to Counteract Age-Related Cellular Dysfunction

**DOI:** 10.3390/genes9030165

**Published:** 2018-03-16

**Authors:** Corina T. Madreiter-Sokolowski, Armin A. Sokolowski, Markus Waldeck-Weiermair, Roland Malli, Wolfgang F. Graier

**Affiliations:** 1 Molecular Biology and Biochemistry, Gottfried Schatz Research Center, Medical University of Graz; Neue Stiftingtalstraße 6/6, 8010 Graz, Austria; markus.weiermair@medunigraz.at (M.W.-W.); roland.malli@medunigraz.at (R.M.); 2 Department of Dentistry and Maxillofacial Surgery, Medical University of Graz; Billrothgasse 4, 8010 Graz, Austria; armin.sokolowski@medunigraz.at; 3 BioTechMed, Mozartgasse 12/2, 8010 Graz, Austria

**Keywords:** mitochondria, aging, caloric restriction, exercise, caloric restriction mimetics, polyphenols, aspirin

## Abstract

Senescence is related to the loss of cellular homeostasis and functions, which leads to a progressive decline in physiological ability and to aging-associated diseases. Since mitochondria are essential to energy supply, cell differentiation, cell cycle control, intracellular signaling and Ca^2+^ sequestration, fine-tuning mitochondrial activity appropriately, is a tightrope walk during aging. For instance, the mitochondrial oxidative phosphorylation (OXPHOS) ensures a supply of adenosine triphosphate (ATP), but is also the main source of potentially harmful levels of reactive oxygen species (ROS). Moreover, mitochondrial function is strongly linked to mitochondrial Ca^2+^ homeostasis and mitochondrial shape, which undergo various alterations during aging. Since mitochondria play such a critical role in an organism’s process of aging, they also offer promising targets for manipulation of senescent cellular functions. Accordingly, interventions delaying the onset of age-associated disorders involve the manipulation of mitochondrial function, including caloric restriction (CR) or exercise, as well as drugs, such as metformin, aspirin, and polyphenols. In this review, we discuss mitochondria’s role in and impact on cellular aging and their potential to serve as a target for therapeutic interventions against age-related cellular dysfunction.

## 1. Introduction

In a rapidly aging society, new treatment options for age-related disorders and diseases will be increasingly important [1,2]. Consequently, in recent decades, research has focused heavily on the processes of aging to reveal potential targets for prolonging health and lifespan [3]. Consistent with this, interventions such as caloric restriction (CR) [3] or exercise [4], as well as pharmacological strategies [5,6] have been well established to improve health and to slow down aging [7]. Aging is linked to the progressive decline of a cell’s or organism’s capacity to counteract stress, damage, and disease [8], resulting in impaired physiological function, pathologies, and death [9]. Cellular hallmarks of aging are shared among various organisms and include genomic instability, telomere attrition, stem cell exhaustion and mitochondrial dysfunction [8,10]. During senescence cells lose their proliferation potential and develop a senescence-associated secretory phenotype (SASP) [11]. Through secretion of pro-inflammatory cytokines, chemokines, and tissue-damaging proteases, among other factors, the SASP has local and systemic pathogenic effects on surrounding cells [12]. Senescent cells also undergo a shift in metabolism, including a change in glycolytic flux and mitochondrial respiration, thereby affecting nearby cells [13].

As adenosine triphosphate (ATP)-producing power plants of the cell, mitochondria are in a unique position to influence an organism’s aging process. Recent reports suggest that mitochondrial function is linked to age-associated biphasic alterations in metabolic activity, including an increase and afterwards progressive decrease in mitochondrial function [3]. In addition, the byproducts of mitochondrial respiration, reactive oxygen species (ROS), are key determinants in the initiation of cellular senescence when present in high concentrations [14]. Moreover, changes in mitochondrial dynamics in fusion and fission, as well as alterations in the mitochondrial membrane potential [15] have been reported to cause cellular dysfunctions during senescence [16]. Hence, changes in mitochondrial Ca^2+^ homeostasis, as well as in endoplasmic reticulum (ER)-mitochondria crosstalk, could reduce the adaptive capacity of cells to withstand stress and increase their vulnerability to age-related diseases [17]. Consequently, it seems reasonable that life-prolonging interventions, such as CR [3] or exercise [4], as well as various drugs [5,6], target mitochondria. Remarkably, reduction of mitochondrial content in vivo has recently been reported to reduce the spectrum of senescence effectors and phenotypes in mice [18]. Nevertheless, since mitochondrial activity changes in a biphasic manner during aging [3], the right interventions need to be set at the right time to successfully counteract age-associated cellular dysfunction instead of triggering it.

Notably, impaired mitochondrial functions were reported to cause accelerated aging that affects primarily organs with high levels of energy demand, such as the brain, the heart, the skeletal muscle, as well as liver and kidney [19]. The critical role of mitochondria in these organs becomes clinically visible in the case of mitochondrial diseases that frequently affect organs with high energy demand, showing clinical features of encephalopathy, dementia, myopathy, exercise intolerance, cardiomyopathy, optic atrophy, liver failure [20], and renal pathologies [21]. In addition, the link between mitochondrial dysfunction and age-related diseases is well-established for Alzheimer’s disease [22], myocardial infarction, and sarcopenia [23]. Notably, the role of mitochondria seems to also be crucial in malignant tumor progression [24], thus, studies that unveil mitochondrial targets, which may serve as potential candidates for new and promising therapeutic strategies against cancer [25], are needed.

This review provides a summary of present knowledge about mitochondria’s unique role in the process of aging and the development of age-related disorders and diseases. Furthermore, the potential of mitochondria to serve as targets for therapeutic interventions against age-related diseases is highlighted.

## 2. Mitochondria 

As descendants of α-proteobacteria, mitochondria are double-membraned and equipped with their own circular genome. After 1.5 billion years of incorporation into the eukaryotic cell, mitochondria are well-integrated and irreplaceable cellular compartments [26]. While the majority of mitochondrial proteins are nuclear-encoded and actively imported into mitochondria [27], the 16.6-kilobase mitochondrial genome includes genetic information for mitochondrial ribosomal and transfer RNA and proteins of the mitochondrial respiration complexes [28]. Since a human requires 65 kg ATP per day on average, mitochondria, as the main producers of cellular ATP, have a Herculean task to fulfill as the most efficient production sites of the cell’s current ATP [29]. 

The protein complexes of the mitochondrial respiration chain are located at the cristae [30]. These infoldings of the inner mitochondrial membrane (IMM) stretch deeply into the matrix, which results in an enormous increase of surface area [31,32]. While the outer mitochondrial membrane (OMM) links mitochondria to other organelles, such as plasma membrane and the ER, and consists of many passive and nonselective transporters, regulating barely the transport of molecules from the cytosol into the intermembrane space (IMS) [33], the IMM has a strongly restricted transport system and a high density of proteins [34]. To fulfill their tasks, mitochondria change their morphology and structure rapidly and continuously undergo fission and fusion [35]. Different proteins are responsible for mitochondrial fusion, including mitofusin 1 (MFN1) and mitofusin 2 (MFN2) in the OMM as well as optic atrophy 1 (OPA1) in the IMM, and for fission, as, for instance, dynamin-related protein 1 (DRP1) [35]. 

To ensure and control mitochondrial Ca^2+^ uptake from the IMS to the mitochondrial matrix, a complex machinery of proteins is located at the IMM [36]. Mitochondrial Ca^2+^ uptake is linked to Ca^2+^ mobilization from the biggest internal Ca^2+^ store, the ER [37], or the entrance of extracellular Ca^2+^ [38]. The close contact between plasma membrane and ER is essential for a process known as store-operated Ca^2+^ entry (SOCE). In this process low Ca^2+^ levels in the ER cause oligomerization and, finally, a conformational change of the Ca^2+^ sensing stromal interaction molecule-1 (STIM1), which then activates the plasma membrane Ca^2+^ channel (ORAI1) through protein-protein interactions, causing Ca^2+^ entry [39]. Cytosolic Ca^2+^ elevations are sensed by mitochondria, which take up Ca^2+^ to buffer cytosolic Ca^2+^ levels in distinct regions of the cytosol, so-called microdomains, and thereby control the activity of Ca^2+^-dependent enzymes [40] and ion channels [41]. Mitochondria are also able to re-shuffle entering Ca^2+^ to the ER [42]. Moreover, the Na^+^/Ca^2+^ exchanger (NCX) and the plasma membrane Ca^2+^ ATPase (PMCA) also regulate cytosolic Ca^2+^ levels by extrusion of Ca^2+^ [43]. 

A finely-tuned complex of proteins ensures and controls the mitochondrial Ca^2+^ uptake, including the pore-forming mitochondrial calcium uniporter (MCU) [44,45], which exists as a hetero-oligomer with its negative regulator mitochondrial calcium uniporter b (MCUb) [46]. Moreover, the essential MCU regulator (EMRE) [47] links the Ca^2+^-sensing proteins mitochondrial Ca^2+^ uptake 1 (MICU1) and mitochondrial Ca^2+^ uptake 2 (MICU2), which control mitochondrial Ca^2+^ homeostasis, to MCU [48]. In addition, several other proteins are involved in MCU-dependent mitochondrial Ca^2+^ uptake, including mitochondrial calcium uniporter regulator 1 (MCUR1) [49], the EF-hand domain containing protein SLC25A23 [50] as well as the uncoupling proteins 2 and 3 (UCP2/3) [51]. Recently, a role for MCU’s cysteine 97 was identified in ROS sensing and the positive regulation of MCU activity, potentially leading to a feedback mechanism for mitochondrial Ca^2+^ uptake [52].

In the mitochondrial matrix, Ca^2+^ ions control the activity of the tricarboxylic acid (TCA) cycle by Ca^2+^-dependent dehydrogenases [53,54]. The reduction equivalents from the TCA cycle, nicotinamide adenine dinucleotide (NADH) and flavin adenine dinucleotide (FADH_2_), donate electrons to the electron transport chain (ECT). Electrons are accepted at the NADH dehydrogenase (complex I) and cytochrome bc1 complex (complex III) and are shuttled via succinate dehydrogenase (complex II), ubiquinone, cytochrome bc1 complex (complex III), and cytochrome *c* to cytochrome *c* oxidase (complex IV), where O_2_ is reduced to H_2_O. The electron transport through complex I to complex IV is coupled to proton pumping from the mitochondrial matrix to the IMS. The electrochemical gradient produced is used for the proton transport from the IMS into the matrix through ATP synthase (complex V), causing a release of energy and phosphorylation of adenosine diphosphate (ADP) to ATP [55]. If electrons are incompletely transferred to complex I or complex III, superoxide anions are generated [56]. To neutralize these potentially harmful byproducts of mitochondrial respiration, mitochondrial manganese superoxide dismutase (MnSOD, SOD2) converts superoxide anions to H_2_O_2_ [57]. While a large portion of H_2_O_2_ may diffuse from the mitochondria to the cytosol, in the matrix of mitochondria several enzymes, like peroxiredoxins (PRDX) 3 and 5, catalase, and glutathione peroxidases 1 and 4 reduce H_2_O_2_ to H_2_O [57]. Notably, H_2_O_2_ is also linked to the NAD and NADP systems and is a key signaling molecule [58]. Hence, reduction of mitochondrial H_2_O_2_ by overexpression of PRDX3 improved glucose tolerance in mice, potentially via inhibition of glycogen synthase kinase-3. Moreover, cells from animals overexpressing PRDX3 had increased resistance to stress-induced cell death and apoptosis [59]. High levels of ROS have harmful effects on their surroundings, including the mitochondrial genome (mtDNA) and proteins, and are, thereby, key determinants in the initiation of cellular senescence [60]. In contrast, low levels of ROS evoke a vaccination-like response, resulting in a strengthened defense shield against ROS, which positively impacts lifespan by increased detoxification of newly-produced ROS [61]. Interestingly, *Mclk1*^+/−^ mutant mice that produce higher levels of ROS than wild-type mice, had an enhanced immune reactivity and slowed aging progression [62]. Moreover, activation of the intrinsic apoptotic pathway by mitochondrial ROS was reported to induce a protective response and improve longevity in *Caenorhabditis elegans* (*C. elegans*) [63].

While mitochondria ensure cell viability by ATP production, they also execute programmed cell death [64]. In response to death-inducing stimuli like metabolic perturbation or oncogene activation, mitochondrial permeability increases. In this regard, the protein OPA1 of the IMM, which tightens cristae junctions by oligomerization, plays a crucial role. The pro-apoptotic BCL-2 family members induce disruption of OPA1 oligomers and thereby widen cristae junctions, causing enhanced permeability [65] and release of second mitochondria-derived activators of caspases (SMACs) and cytochrome *c* into the cytosol through a mechanism yet to be described [66]. By deactivating proteins that inhibit apoptosis (IAPs), SMACs indirectly activate caspases. In addition, cytochrome *c* binds to apoptotic protease activating factor 1 (APAF1) and ATP, forming the apoptosome complex, which cleaves pro-caspase 9 into its active form caspase-9, and thereby activates effector caspases [67]. During senescence this process of programmed cell death is less tightly regulated, leading to accumulation of dysfunctional and damaged cells [68]. 

Since all of these mitochondrial tasks are highly complex and need to be well-balanced and controlled, numerous targets may be affected by aging or impacted by dysregulation of the process of aging.

## 3. Mitochondrial Changes during Aging

### 3.1. Mitochondrial Ca^2+^ Homeostasis

During the process of aging, the protein machinery controlling mitochondrial Ca^2+^ uptake and homeostasis undergoes remarkable changes. For instance, the MCU channel is oxidized by ROS, resulting in increased MCU channel activity, which, in turn, further elevates mitochondrial ROS levels and increases the risk for mitochondrial Ca^2+^ overload-induced cell death [52]. Moreover, the expression of MCU, as well as the crosstalk between ER and mitochondria was increased in long-term cultured rat hippocampal neurons, leading to elevated mitochondrial Ca^2+^ levels, while store-operated Ca^2+^ entry was inhibited via downregulation of STIM1 and ORAI1 in these aging neurons [69]. In line with that, replicative senescence and oncogene-induced senescence caused mitochondrial Ca^2+^ accumulation by inositol 1,4,5-trisphosphate receptor type 2 (ITPR_2_)-triggered Ca^2+^ release from the ER and mitochondrial Ca^2+^ uptake via MCU in human mammary epithelial cells (HMEC). In turn, loss of ITPR_2_ and MCU counteracted the development of senescence [70]. Mitochondrial Ca^2+^ overload, as well as high levels of ROS promotes cell death by collapse of the mitochondrial membrane potential and opening of the mitochondrial permeability transition pore (mPTP) [71]. Moreover, age-associated loss of mitochondrial membrane potential [15] might further increase the risk of mPTP opening [71]. Release of metabolites such as ROS, Ca^2+^, NAD^+^, and glutathione into the cytosol was shown to disrupt cellular homeostasis and to increase oxidative damage [72] and possibly cause a decline in mitochondrial function. Indeed, mitochondrial Ca^2+^ flux in response to cytosolic Ca^2+^ was reported to decrease progressively in *C. elegans* during aging [73]. The importance of sufficient mitochondrial Ca^2+^ uptake was demonstrated in mice lacking Fus1, a small mitochondrial protein regulating mitochondrial Ca^2+^ homeostasis. Loss of Fus1 caused inefficient accumulation of Ca^2+^ in mitochondria and decreased respiratory reserve capacity, resulting in the decreased lifespan of mice with knockout of Fus1 [74]. These partly conflicting results suggest that fine-tuning of mitochondrial Ca^2+^ homeostasis during aging is a tightrope walk between meeting mitochondria’s demand for Ca^2+^ [74] and triggering harmful processes like increased ROS production or mPTP opening by Ca^2+^ overload [74].

### 3.2. Mitochondrial Respiration, Reactive Oxygen Species Production, and Reactive Oxygen Species Defense

Aging was typically associated with decreased mitochondrial metabolism [10]. In apparent contradiction, approaches to extend lifespan are frequently linked to a reduction in energy intake and decreased overall energy production [75]. The hypothesis about a biphasic regulation of metabolism during aging developed in response, suggesting increased mitochondrial activity in middle-age followed by constant decline in advanced age [3]. In support of this, TCA metabolites changed in a biphasic manner in rhesus monkeys, including a decrease in the NAD^+^/NADH ratio at middle-age (15–16 years) and elevation in increased age (28–32 years) [76]. Moreover, since changes in mitochondrial Ca^2+^ levels mirrored those in energy production—initially elevated [52,69] before they declined as a result of mitochondrial Ca^2+^ overload-induced damage [72]—it seems safe to assume that mitochondrial respiration is boosted at middle-age. Since increased mitochondrial respiration is linked to elevated levels of ROS, enhanced mitochondrial respiration at middle age may promote cellular damage by ROS [3]. Therefore, a well-developed antioxidant defense shield might be necessary to counteract ROS-induced cellular dysfunction [61]. In this regard, a study with transgenic mice overexpressing human catalase in peroxisomes, nucleus, or mitochondria highlighted the importance of mitochondrial antioxidant defense mechanisms, showing that median and maximum life span were maximally increased in mitochondrial catalase-overexpressing animals [77]. Another study failed to see a positive impact of catalase overexpression or combined overexpression of MnSOD with cytosolic copper-zinc superoxide dismutase CuZnSOD, as well as of catalase with CuZnSOD on the lifespan of mice. Notably, catalase overexpression in this transgenic mouse model occurred in peroxisomes [78]. Interestingly, overexpression of cytosolic thioredoxin 1 was reported to extend mainly the earlier part of murine lifespan, but not to affect maximum life span [79]. It seems likely that the ROS-defense system might be successful just to a certain extent and simply delay the onset of mitochondrial respiration dysfunction and decline, possibly differing within the various species. In old normal human fibroblasts (NHFs), oxygen consumption rate (OCR), and ATP levels significantly increased, while glycolytic flux and lactate levels decreased [13]. In contrast, in murine brain and liver the content and activity of critical enzymes of mitochondrial function, including complex I and complex III, as well as MnSOD, decreased with age. Decline was detected after 28 weeks and progressed further until 92 weeks [80]. Consistent with this, brain mitochondria from aging mice (16–24 months) exhibited decreased F_1_F_O_ ATP synthase activity and defective F_1_F_O_ complex coupling, which may be caused by the mitochondrial protein cyclophilin D [81]. Moreover, in cardio myocytes of middle-aged rats (two years) the efficiency of the creatine kinase phospho-transfer pathways declined during senescence, which resulted in reduced oxidative phosphorylation (OXPHOS), decreased affinity of mitochondrial ADP and, ultimately, decreased heart muscle performance [82]. In summary, studies from various cell types and animals in different studies do not allow a clear conclusion yet but make further studies necessary with regard to a potentially biphasic metabolic change during aging. 

### 3.3. Mitochondrial Genome and Unfolded Protein Response

Mutations of mitochondrial DNA (mtDNA) are estimated to be 10- to 17-fold higher than in the nuclear genome [83]. In line with the free radical theory of aging overwhelming ROS production is the main cause for this damage to the mitochondrial genome, which is especially susceptible to this type of genomic damage due to a lack of histones and limited DNA repair mechanisms [84]. Consequently, a well-developed antioxidant defense offered protection from mitochondrial dysfunction and resulted in greater longevity in various species [84]. In addition, antioxidants counteracted cell cycle arrest and aging, induced by mtDNA double-strand breaks, in mice [85]. Studies in conplastic mouse strains further highlighted the crucial role of mtDNA, demonstrating that different mtDNA variants are able to promote alterations in mitochondrial function and cellular adaptive responses which impact metabolic performance and aging [86]. Point mutations in mtDNA significantly accumulated in brain mitochondria of aged mice and caused mitochondrial dysfunction by altering the OXPHOS machinery [87]. However, a high percentage of mutated mtDNA is necessary to induce dysfunction and diseases [88]. Levels of mtDNA in mouse liver and uterus decreased significantly during aging and mtDNA copy numbers were reduced in aged oocytes [89]. Notably, high levels of mtDNA deletions were reported for specific regions of the human brain [90] and substantia nigra neurons [91,92]. Moreover, an accumulation of mtDNA deletions was also found in aged human skeletal muscle fibers [93]. Since mtDNA encodes essential parts of the OXPHOS machinery, deletion of mtDNA causes OXPHOS dysfunction and a decline of cellular function [88]. The induction of mitochondrial unfolded protein response (UPR^mt^) was suggested as a possible mechanism to tolerate mtDNA deletions to a certain extent [88]. UPR^mt^ is a protective transcriptional program from the cells to sense and respond to mitochondrial dysfunction by inducing mitochondrial protein homeostasis and regeneration of defective mitochondria [94]. In a heteroplasmic *C. elegans* strain, containing wild-type mtDNA and mtDNA lacking four essential genes, UPR^mt^ was constitutively activated by dysfunctional OXPHOS. Moreover, even deleterious mtDNA was maintained by UPR^mt^, possibly in an attempt to recover OXPHOS activity by promoting mitochondrial biogenesis [88]. It was shown in *C. elegans* that OXPHOS dysfunction induces the activating transcription factor associated with stress (ATFS1), which binds directly to the OXPHOS gene promotors in the nuclear and mitochondrial genomes and thereby promotes OXPHOS recovery during the UPR^mt^ [95]. In mammals the transcription factor ATF5 was found to induce similar transcriptional responses as ATFS1 in *C. elegans* and even rescued UPR^mt^ signaling in ATFS1 deficient worms [94]. Furthermore, in mammalian cells, it was shown that mitochondrial protein folding stress activates chaperone availability and reduces protein synthesis in the matrix via translational inhibition. The crucial impact of UPR^mt^ on the process of aging was demonstrated in worms, in which UPR^mt^ delayed aging and promoted life span via ATFS1 [96]. Recently, UPR^mt^ was also shown to prolong the lifespan of *Drosophila melanogaster* by activation of the forkhead protein O (FOXO), which induces a set of chaperones implicated in lifespan extension [97].

### 3.4. Mitochondrial Mass and Structure

The main transcription factor of UPR^mt^ in nematodes, ATFS1, controls mitochondrial biogenesis to compensate for loss of OXPHOS activity [95]. Consequently, mitochondrial mass might be increased in the case of UPR^mt^ activation during aging [98]. Indeed, a progressive accumulation of mitochondria during the process of aging was described for the nematode *C. elegans* and linked to age-related decline in so-called mitophagy [99]. After mitochondria are replicated via symmetric fission, they are in a constant fusion and fission process. However, damage occurs over time or as a consequence of stress and the defective mitochondria are removed via a specific degradation pathway, mitophagy, to keep the host cell alive [100]. For efficient mitophagy, an intact fission complex is required [101]. Notably, in neurons from aged rhesus macaques, a phenomenon called *mitochondria-on-a-string* (MOAS), characterized by thin segments of mitochondria mixed with large ones, a phenotype already described for neurodegenerative diseases, was found and linked to incomplete mitochondrial fission due to dysregulation of the fission machinery [102]. Short-term induction of mitochondrial fission in midlife by DRP1overexpression enhanced mitophagy in *D. melanogaster.* A shift towards accumulation of dysfunctional elongated mitochondria was thereby prevented and health, as well as lifespan, improved significantly [103]. Notably, increased levels of mitochondrial fission proteins FIS1 and DRP1 were also reported for middle-aged mice (15 months) [104]. Nevertheless, the function of FIS1 in mitochondrial fission of human cells is questioned by findings demonstrating that FIS1 is not required for fission, but for the disposal of defective mitochondria [105]. Consistent with this finding, FIS1 was also reported as an essential part of the mitophagy process [106]. However, enhanced mitochondrial fission might be a defense mechanism of mitochondria in middle-age, but mitochondria’s ability to alter the structure in order to control function might be exhausted in old age. For instance, mitochondrial fusion was induced by the increased activity of MFN1and OPA1 in old NHFs [13]. Moreover, an expression decline of MFN1, MFN2, OPA1, and DRP1 caused age-related muscle loss in humans and mice, which could be counteracted by lifelong regular exercise [107]. Acute depletion of OPA1 resulted in mitochondria of smaller size, mitochondrial dysfunction, and ER stress, inducing muscle loss and aging [107]. In long-lived *D. melanogaster* (10–12 weeks), mitochondria showed cristae vacuolization and the distribution of functional proteins such as ATP synthase was hampered [108]. Another study also confirmed a disruption of the IMM organization in old *D. melanogaster,* characterized by the lack of well-developed cristae and cristae junctions and positively correlating with the rate of mitochondrial respiration [109]. In contrast, the changes in mitochondrial ultrastructure were less apparent and strongly tissue-specific in aged mice. Mitochondria of mouse liver exhibited the strongest age-specific phenotype, which was characterized by a central matrix void, but did not result in a reduction of OXPHOS [109]. In cardiomyocytes of 24-month old rats the number of mitochondrial cristae was markedly reduced, their parallel position was lost, and they did not fill the entire mitochondrial matrix. Consistent with this finding, the surface area of the IMM was reported to be decreased in aged rats [110]. In yeast, age-associated accumulation of sphingoid bases, aliphatic amino alcohols, including sphingosine, caused fragmentation of the mitochondrial network, resulting in impaired mitochondrial respiration, loss of mitochondrial membrane potential, and a decline in lifespan [111]. In summary, these recent reports demonstrate that alterations of mitochondrial structure during aging have wide-ranging effects on mitochondrial and cellular function, but might offer unique targets to manipulate mitochondrial function.

## 4. Targeting Mitochondria to Counteract Aging

### 4.1. Behavioral Interventions

Caloric restriction: From *C. elegans* [112] to primates [113], caloric restriction (CR) has been shown to counteract age-related decline and to increase lifespan [114,115]. The occurrence of metabolic, neurological, and cardiovascular diseases was decreased by a 20–40% reduction in caloric intake [116]. While reducing the overall metabolism, CR was reported to shift the metabolism from carbohydrate to fatty acid metabolism, strongly stimulating mitochondrial energy production [75]. CR also enhanced the activity of complex IV as well as ROS-producing complexes I and III in the brains of mice, causing elevated MnSOD activity and redox capacity in CR brains [117]. Consistent with that, low levels of mitochondrial ROS were reported to initiate health-promoting antioxidant defense mechanisms and increase life span via so-called “mitohormesis” [118]. The effect of CR is not just limited to mitochondrial respiration. In liver mitochondria of mice, Ca^2+^ retention capacity was enhanced strongly by CR in comparison to ad libitum diet, offering protection from ischemia/reperfusion damage, a condition frequently observed in age [119]. Additionally, CR decreased the activity of cyclophilin D through deacetylation, resulting in the increased capacity of mitochondria to buffer Ca^2+^ and enhanced tolerance against excitotoxicity in rat brain mitochondria [117]. In yeast, CR optimized the signaling pathway of SNF1, the yeast orthologue to AMP-activated protein kinase (AMPK), thereby regulating cellular energy homeostasis and extending life span [120]. Moreover, short-term CR, as well as CR mimetic drugs like metformin or resveratrol, activated AMPK-mechanistic target of rapamycin (mTOR) signaling in aging kidney tissues, as well as in human primary proximal tubular cells [121]. AMPK was shown to stimulate sirtuin 1 (SIRT1) via an increase in NAD^+^ levels [122] and to induced mitochondrial biogenesis [123]. Indeed, CR increased expression of SIRT1 and transcription factor a (TFAM), directly stimulating mtDNA replication and transcription, and resulted in increased mitochondrial content and function in skeletal muscle of healthy, non-obese humans [124]. Nevertheless, the impact of CR on mitochondrial function in humans during aging was questioned by a recent study showing that 12 months of a 25% CR did not affect muscle mitochondrial function and oxidative stress in healthy, non-obese humans. Only individuals with a higher ATP flux/O_2_ ratio at baseline benefited from CR in regard to muscle mitochondrial ATP synthesis rates and coupling [125]. These contradictory results might suggest that the right timing determines the outcome of CR, as well as the individual’s ability to react to this intervention. 

Exercise: In a number of species, age-related muscle loss is linked to impaired mitochondrial biogenesis and function, as well as to decreased metabolic capacity [126]. Proliferator-activated receptor γ co-activator 1α (PGC-1α) crucially contributes to mitochondrial biogenesis by activating nuclear respiratory factors (NRF1 and NRF2), which promote expression of nuclear-encoded mitochondrial proteins, and TFAM [127]. In old mice, PGC-1α and mitochondrial sirtuin 3 (SIRT3) were downregulated [128]. Notably, exercise normalized age-related changes of these proteins and, in addition, the expression of catalase, was enhanced by exercise, potentially contributing to improved resilience of arteries in exercising mice by elevated ROS defense mechanisms [128]. Additionally, in muscles of aged rats, PGC-1α and TFAM were found to be downregulated and, in line, mtDNA content was decreased. Interestingly, the aging-related decline of the PGC-1α signaling pathway could be reversed by exercise, increasing levels of PGC-1 α, TFAM and the mtDNA content. These training effects were linked to increased expression of AMPK, p38 mitogen-activated kinase (MAPK) and SIRT1, potentially functioning as up-stream targets of exercise [127]. In addition to mitochondrial biogenesis, mitochondrial Ca^2+^ homeostasis has also been shown to be affected during aging, revealing downregulation of MCU in aged mice [4]. Analysis of muscle biopsies from 70-year old subjects revealed changes in proteins involved in mitochondrial Ca^2+^ uptake and in mitochondrial shape by nine weeks of physical training or neuromuscular electrical stimulation. Improved muscle function and structure by both types of training were linked to increased protein levels of MCU. Interestingly, electron microscopy revealed remodeling of mitochondrial shape in muscles trained by electrical stimulation, which was potentially mediated by an increased expression of cristae structure-shaping protein OPA1 [4]. Interestingly, endurance exercise also normalized redox homeostasis and induced a change in the subcellular localization of p53 to mitochondria versus nucleus, which was linked to beneficial effects on mitochondrial function and maintenance of mtDNA stability [129].

### 4.2. Pharmacological Strategies

*Polyphenols*: Polyphenols, such as resveratrol (RSV) or green tea polyphenols, were shown to extend lifespan in *Saccharomyces cerevisiae* [130], *C. elegans* [131], *D. melanogaster* [132], fish [133], and mice [134]. Moreover, the low incidence of coronary heart disease in French people despite the rather high intake of dietary cholesterol and saturated fat, known as the *French paradox*, was hypothesized to be due to frequent consumption of RSV-rich red wine [135]. Clinical trials confirmed that RSV is safe and well-tolerated, but also demonstrated the very poor bioavailability of RSV [136]. The low bioavailability of RSV in combination with its low approximate concentration in red wine (0.1–14.3 mg/L) [136], as well as the enormous toxic effects induced by ethanol, makes red wine unlikely to serve as a fountain of youth [137]. However, recent studies suggest that high intake of fat is even associated with lower total mortality, while high carbohydrate intake was linked to a higher risk of total mortality [138]. Thus, the hypothesis about a crucial role of RSV’s in the *French paradox* has to be reevaluated.

Nevertheless, RSV was found to mimic features of CR, such as glucagon and catecholamine release, boosting cyclic adenosine monophosphate production, which, in turn, activates cell-type dependent effectors and leads to elevated Ca^2+^ levels and activation of the CaMKKβ-AMPK pathway [139]. Enhanced AMPK activity was reported to stimulate SIRT1 via an increase in NAD^+^ levels [122]. Other reports claimed that RSV directly activates SIRT1 [140,141], causing enhanced mitochondrial biogenesis [142] and altered metabolism [5]. Moreover, RSV was also reported to trigger mitochondrial respiration and ROS production at low concentrations [143]. Low levels of ROS might stimulate AMPK activity [144] as well as the development of antioxidant defense mechanisms, which might, in turn, result in extended lifespan [145]. Nevertheless, the individual ability to develop defense mechanisms against ROS might be necessary for RSV to impact age-related dysfunctions and diseases positively [143]. For instance, in an Alzheimer’s disease model, RSV failed to reduce elevated H_2_O_2_ production, whereas the ubiquinone derivative, MitoQ, an electron scavenger preventing mitochondrial ROS formation [146], could normalize H_2_O_2_ levels under this condition [147].

*Antioxidants*: In addition to MitoQ, the Murphy lab has synthesized several other mitochondria-targeted antioxidants by covalently attaching lipophilic triphenylphosphonium cations to endogenous antioxidants, including *α*-tocopherol (MitoVitE) and the synthetic spin trap compound α-phenyl-tert-butylnitrone (MitoPBN) [148]. Furthermore, they created the mitochondrial-targeted piperidine nitroxide MitoTEMPOL [149]. Due to mitochondrial accumulation, these compounds exhibited more enhanced protective effects than untargeted analogs [148]. In rat brains, MitoQ suppressed peroxynitrite-mediated mitochondrial function induced by lead toxicity [150]. Furthermore, MitoQ was renoprotective in diabetic mice, possibly via an action of uncoupling [151]. In addition, oral administration of MitoQ reversed aortic stiffness in old mice [152]. MitoQ was even approved for use in humans. Twenty-eight days of oral MitoQ (40 or 80 mg/day) supplementation decreased liver damage in a phase II study of hepatitis C patients [153]. Moreover, the novel mitochondrial-targeted antioxidant elamipretide (SS-31), whose mode of action is still under investigation [154], was successfully tested in a clinical trial about heart failure treatment [155] and is currently under trial as a potential therapy of mitochondrial diseases [156]. All of these results prove that an imbalance in ROS signaling plays a crucial role in various diseases and aging. 

Nevertheless, the relevance of antioxidants in preventing age-related cellular dysfunction by the reduction of oxidative stress was challenged by the finding that small levels of ROS provoke a vaccination-like stimulus, thus causing development of antioxidant defense mechanisms and a positive impact on lifespan [118]. Consequently, researchers have suggested an inverted U-shaped dose-response relationship between ROS levels and lifespan. In support, the three antioxidants N-acetylcysteine, vitamin C and RSV lengthened lifespan at low concentrations and shortened lifespan at high concentrations in *C. elegans* [157]. The difficulty in finding the right dosage might explain controversial outcomes of clinical trials regarding antioxidant supplementation therapies in humans [158]. In animals, as well, no conclusive statement regarding the success of treatment with antioxidants is possible. Administration of MitoQ to 24-month-old mice for 15 weeks did not reduce mitochondria ROS levels or normalize mitochondrial membrane potential and respiration in muscle tissues. Moreover, there was no effect on lifespan [159]. In contrast, in mice with mtDNA mutations, which accelerate aging, the mitochondria-targeted antioxidant SkQ1 counteracted increased mitochondrial ROS levels and improved health and lifespan [160]. In the case of aging, it might be difficult to find the right dosage and to identify the therapeutic window in which treatment with antioxidants may be beneficial, since the individual ROS defense mechanisms is likely to undergo changes, possibly in a biphasic modulation [3]. 

*Aspirin*: Long-term usage of acetylsalicylic acid (Aspirin^®^), a nonsteroidal anti-inflammatory drug, was shown to slightly increase the lifespan of worms [161], flies [162], and mammals [163]. After oral administration, aspirin is metabolized to salicylate [6]. This metabolite was shown to activate AMPK in HEK-293 cells directly, critically influencing cell growth and metabolism. Accordingly, fatty acid oxidation, associated with increased phosphorylation of AMPK, was stimulated by salicylate in isolated hepatocytes of mice [6]. Additionally, in *C. elegans*, aspirin treatment increased the expression of lipid hydrolysis and fatty acid β-oxidation-related genes, which activated DAF-12 and DAF-16, the worm orthologue to FOXO, and extended lifespan [161]. Nevertheless, despite reducing the risk for colorectal cancer [164] and potentially contributing to cardioprotection [165,166], the successful use of aspirin in humans to increase health and lifespan has yet to be tested in a clinical trial and is rather questionable due to a simultaneously increased risk of gastrointestinal bleeding [167].

*Omega-3 fatty acids*: Treatment with ω-3 fatty acid α-linolenic acid (ALA) increased lifespan of *C. elegans* via the activation of the transcription factors NHR-49, the worm orthologue of human PPARα, and SKN1, the worm orthologue of human NRF2. NHR-49-activated genes are involved in the β-oxidation of free fatty acids, promoting the generation of energy via mitochondrial respiration and NRF2-induced mitochondrial biogenesis [168]. In the brains of aged mice, exhibiting reduced ATP levels and decreased activity of complexes I, II and IV of the mitochondrial respiration system, ω-3 polyunsaturated fatty acids from fish oil induced neuroprotective actions and improved mitochondrial ATP production and function [169]. Consistent with these findings, ω-3 polyunsaturated fatty acids increased mitochondrial protein synthesis and significantly reduced mitochondrial oxidative stress in aged humans (65–85) [170]. Nevertheless, conclusive clinical trials about the impact of ω-3 fatty acids on health and lifespan of humans are still missing and, consequently, their potential as anti-aging drugs is elusive.

*Metformin*: Metformin, a biguanide used for diabetes prevention and management for 60 years, was reported to display greatly beneficial effects on aging in cellular and animal models, as well as in pilot human studies, far beyond that expected from improving glycemic control [1]. Despite its successful use in the treatment of type-2 diabetes by glucose-lowering and insulin-sensitizing effects, the mode of action of this drug remains largely elusive. One reason for its therapeutic actions might be the inhibition of mitochondrial respiration by reducing the activity of complex I and ATP synthase as well as by stimulating ROS production [171], possibly ending in a process of mitohormesis [118]. That metformin drastically affects metabolism, was further highlighted by the finding that metformin effectively reduces acetyl coenzyme A (CoA) synthesis [172]. Moreover, reduction in mitochondrial respiratory capacity resulted in inactivation of mechanistic target of rapamycin complex 1 (mTORC1), which inhibited growth via the mitochondrial β-oxidation regulator acyl-CoA dehydrogenase family member-10 (ACAD10). Accordingly, ACAD10 was necessary for the lifespan-extending effect of metformin in *C. elegans* [173]. As a caloric-restriction mimetic, metformin was believed to inhibit mTORC1 through AMPK by increasing the cellular AMP/ATP ratio. However, inhibition of mTORC1 signaling was even found in the absence of AMPK [174] and inhibition of mTORC1 down-regulated nuclear-encoded mitochondrial genes, resulting in decreased mitochondrial biogenesis and mitochondrial respiration [175]. Interestingly, the sensitivity of cells to metformin is strongly dependent on their ability to cope with energetic stress [176]. Consequently, metformin might induce a senolytic effect [177], causing specific cell death in aged cells with decreased compensatory mechanisms to counteract changes in cellular metabolism. 

*Senolytics*: Senolytic drugs were reported to induce apoptosis of senescent cells, thus preventing the local and systemic pathogenic effects of senescent cells conveyed by the release of proinflammatory cytokines and chemokines, tissue-damaging proteases and inhibition of stem and progenitor cell function [12]. By hosting various proteins of the apoptosis machinery, mitochondria are in a unique position to mediate the effects of these drugs [64]. Accordingly, the first senolytic agents discovered all affected mitochondria, including dasatinib, quercetin, and navitoclax [12]. The cancer drug dasatinib, a tyrosine kinase inhibitor approved for treatment of chronic myeloid leukemia, was reported to regulate mitochondrial biogenesis via increased expression of PGC-1α [178] and to increase mitochondrial ROS production drastically [179]. Navitoclax, in use to treat various types of cancer, targets BCL-2 proteins and thereby specifically kills cancer and senescent cells [180]. Moreover, as in RSV, the polyphenol quercetin was shown to modulate mitochondrial function in a concentration-dependent manner [181]. Therefore, the specific metabolic pattern, as well as the ability to compensate for changes in metabolism during aging, might be crucial for the compounds’ mode of action. However, researchers have not yet specifically investigated how these compounds work, nor how to recognize the right timing for an intervention.

## 5. Conclusions

**Figure 1:**
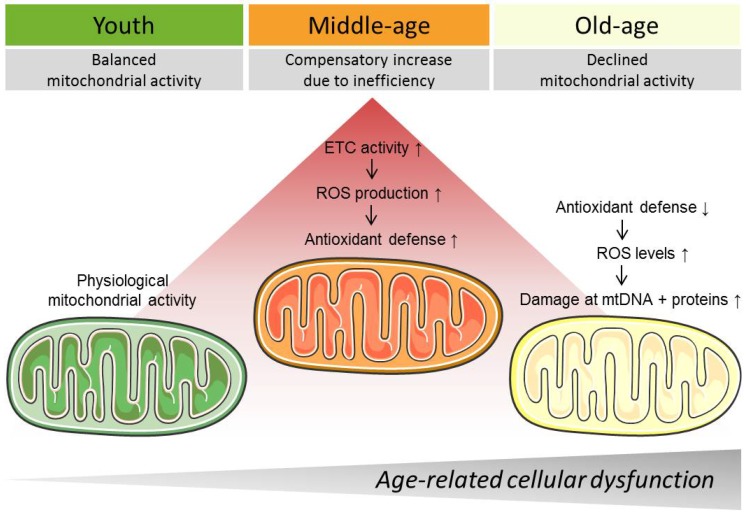
Biphasic alterations of mitochondrial function during aging. ETC: Electron transport chain, ROS: Reactive oxygen species, Mito: Mitochondrial, MtDNA: Mitochondrial desoxyribonucleic acid. Created using Servier Medical Art (Les Laboratoires Servier, Suresnes, France).

The process of aging evokes various alterations in mitochondrial Ca^2+^ handling [17], mitochondrial respiration [80], mitochondrial structure [13], as well as in the mitochondrial genome [84], which are mutually interrelated to each other. Results from cell culture and animal experiments suggest enhanced mitochondrial activity in middle age, but a decline in old age [3]. Initially, increased activity of mitochondria might compensate for the decreased mitochondrial efficiency that occurs during aging. However, this enhanced mitochondrial activity might harm the cell long-term, for instance, by increased ROS production, and might even further promote age-related cellular dysfunction (Figure 1). Interventions, like CR [117], exercise [128], and drugs, such as polyphenols [139], antioxidants [160], metformin [171], ω-3 fatty acids [168], aspirin [6], and senolytics [12], specifically target mitochondria and thereby successfully counteract these age-related damages. Nevertheless, it seems critical when intervention is brought to bear and at which time. Accordingly, clinical studies often yield inconclusive results with regard to anti-aging interventions [158]. 

Consequently, it is of major importance to further investigate the molecular processes behind the role of mitochondria in aging, as well as their potential to serve as targets for therapeutic interventions.
